# The challenges of diagnosing osteoporosis and the limitations of currently available tools

**DOI:** 10.1186/s40842-018-0062-7

**Published:** 2018-05-29

**Authors:** Palak Choksi, Karl J. Jepsen, Gregory A. Clines

**Affiliations:** 1 Division of Metabolism, Endocrinology & Diabetes, Department of Internal Medicine, University of Michigan, Ann Arbor, MI USA; 2 Departments of Orthopaedic Surgery and Biomedical Engineering, University of Michigan, Ann Arbor, MI USA; 3 Endocrinology Section, Ann Arbor VA Medical Center, 2215 Fuller Road, Research 151, Ann Arbor, MI 48105-2399 USA

**Keywords:** Osteoporosis, Dual X-ray absorptiometry, Peripheral quantitative computed tomography, Skeletal fracture, Skeletal biomechanics, Bisphosphonates, Denosumab, Teriparatide, Romosozumab

## Abstract

Dual-energy X-ray absorptiometry (DXA) was the first imaging tool widely utilized by clinicians to assess fracture risk, especially in postmenopausal women. The development of DXA nearly coincided with the availability of effective osteoporosis medications. Although osteoporosis in adults is diagnosed based on a T-score equal to or below − 2.5 SD, most individuals who sustain fragility fractures are above this arbitrary cutoff. This incongruity poses a challenge to clinicians to identify patients who may benefit from osteoporosis treatments. DXA scanners generate 2 dimensional images of complex 3 dimensional structures, and report bone density as the quotient of the bone mineral content divided by the bone area. An obvious pitfall of this method is that a larger bone will convey superior strength, but may in fact have the same bone density as a smaller bone. Other imaging modalities are available such as peripheral quantitative CT, but are largely research tools. Current osteoporosis medications increase bone density and reduce fracture risk but the mechanisms of these actions vary. Anti-resorptive medications (bisphosphonates and denosumab) primarily increase endocortical bone by bolstering mineralization of endosteal resorption pits and thereby increase cortical thickness and reduce cortical porosity. Anabolic medications (teriparatide and abaloparatide) increase the periosteal and endosteal perimeters without large changes in cortical thickness resulting in a larger more structurally sound bone. Because of the differences in the mechanisms of the various drugs, there are likely benefits of selecting a treatment based on a patient’s unique bone structure and pattern of bone loss. This review retreats to basic principles in order to advance clinical management of fragility fractures by examining how skeletal biomechanics, size, shape, and ultra-structural properties are the ultimate predictors of bone strength. Accurate measurement of these skeletal parameters through the development of better imaging scanners is critical to advancing fracture risk assessment and informing clinicians on the best treatment strategy. With this information, a “treat to target” approach could be employed to tailor current and future therapies to each patient’s unique skeletal characteristics.

## Background

Two million osteoporosis fractures occur in the U.S. each year costing approximately $19 billion [1]. Despite the medical and economic costs of fragility fractures, osteoporosis screening is often overlooked and viewed as a low priority. Dual-energy X-ray absorptiometry (DXA) was introduced in the mid-1980s as a rapid and safe imaging modality to estimate bone mineral density (BMD) and predict skeletal fracture risk [2]. Up until the widespread use of DXA, patients at high fracture risk were not easily identified and effective osteoporosis medications were limited. Today, not only are DXA scanners utilized in hospital radiology departments but they are also found at many physician group outpatient clinical practices.

The World Health Organization (WHO) defines osteoporosis as a BMD T-score of − 2.5 or lower at any one location or having a previous fragility fracture. The rationale for choosing this T-score was that the proportion of postmenopausal women with a T-score less than − 2.5 is equal to the fragility fracture lifetime risk of 30% [3]. It was expected that individuals who were below this T-score would have a greater fracture risk. Further, this cutoff value of − 2.5 was expected to change over time as the accumulation of experience and data would provide insight into a more appropriate cutoff value. However, this cutoff value has not changed in over 25 years despite data indicating that the T-score of − 2.5 captures only approximately 50% of women with fragility fractures [4]. There is less consensus of the definition of osteoporosis in men. The WHO, however, recommends similar T-score thresholds in men who are greater or equal to 50 years of age [5]. Because of a larger skeletal structure, fracture risk for men is less than in women for any similar T-score; and the fracture risk in men is less than half of women starting at age 55 [6]. Even though fracture rates are less than in men, the mortality associated with fractures is significantly higher [7, 8].

Thus, individuals with a T-score below the − 2.5 cutoff may be at higher risk of fracturing but they do not account for the majority of fracture cases in either women or men [9, 10]. While one of the challenges in management is to avoid over-treatment, individuals with T-scores above − 2.5 with other risks for fracture deserve attention, and should qualify for appropriate treatment as well.

Other commonly used methods to predict fracture risk such as the FRAX scoring system, trabecular bone score and bone turnover markers may provide an incremental improvement in risk assessment when combined with DXA. Ultimately, skeletal biomechanics that include size, shape and bone molecular structure are the predictors of bone strength. Understanding how each of these variables affects the skeleton is critical in the development of better fracture prediction tools to accurately identify those at a high risk for fractures.

## Bone biomechanics

**Fig. 1:**
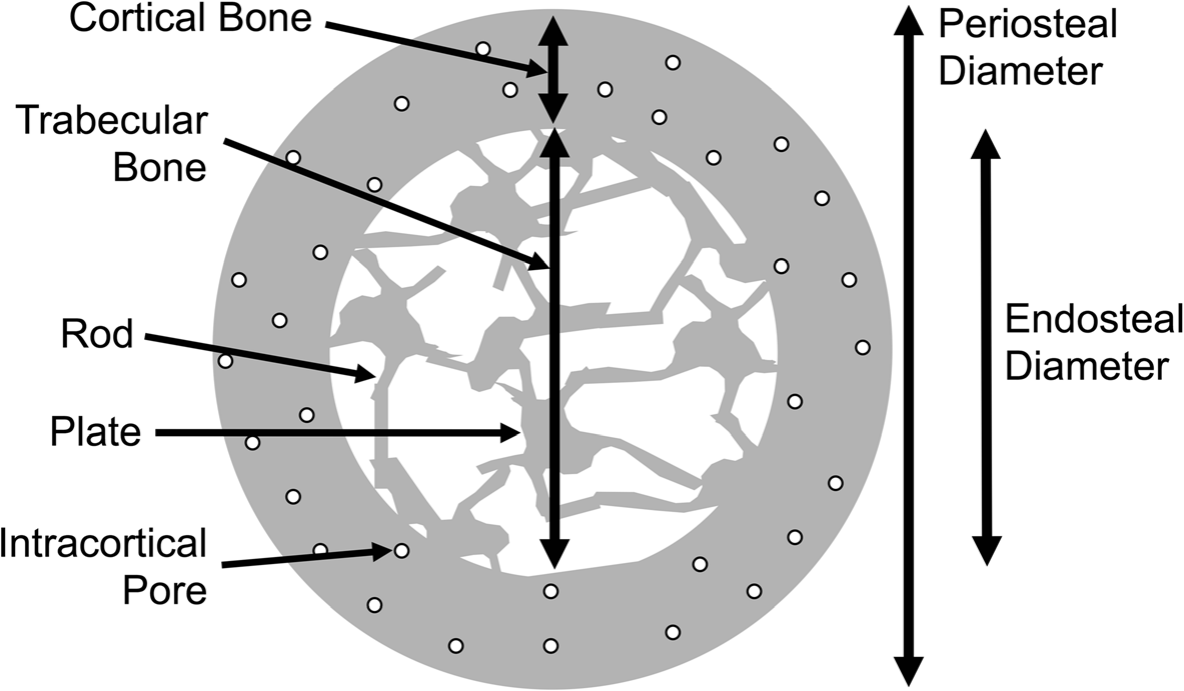
Structural characteristics of bone. Bone is comprised of a dense cortical shell that surrounds a spongy trabecular bone network. The periosteal diameter combined with the endosteal diameter determines cortical thickness. The size of bone along with cortical thickness and porosity significantly contribute to bone strength. The inner trabecular compartment contains a network of plates and rods that also contribute to bone strength

The adult skeleton is composed of 206 uniquely shaped structures, each of which coordinately adapts its morphology and tissue-level material properties to support the physiological loads encountered during daily activities. Cortical bone is the dense outer shell that is divided into three surfaces: the periosteum, intracortical pores, and endosteum (Fig. 1). Trabecular bone is surrounded by cortical bone and is comprised of a spongy network of connected plates and rods. To remain strong but light, the system uses cortical bone in the diaphyses and trabecular bone surrounded by a relatively thin cortical shell in the metaphyseal regions. The proportion of cortical and trabecular bone varies depending on the location. For example, the ultradistal radius is approximately 25% cortical and 75% trabecular bone. The 1/3 proximal radius is primarily all cortical bone.

**Fig. 2:**
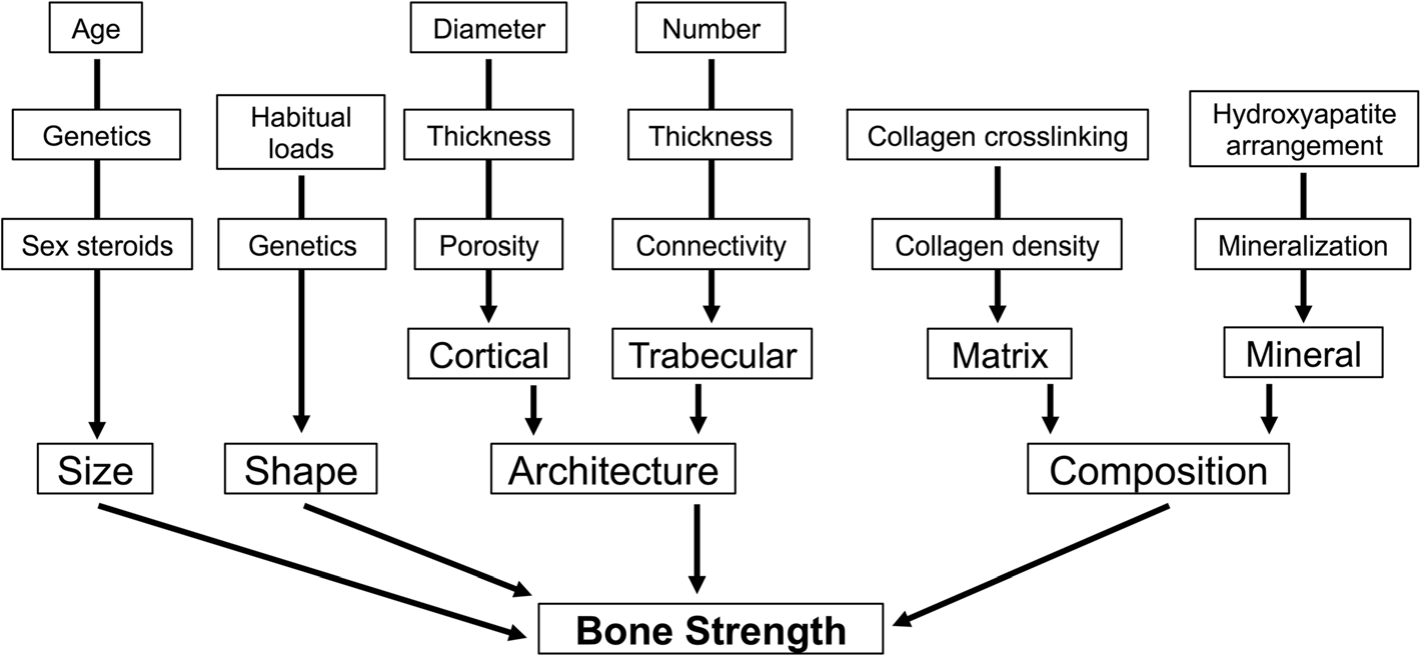
Determinants of bone strength. Bone strength is a composite summation of numerous skeletal characteristics. The size of bone increases with age and with puberty. Ultimate bone size also has a large genetic contribution. Genetics and habitual loading determine bone shape. The architecture of bone is a complex interplay among many structural components. Cortical diameter, thickness and porosity contribute to cortical strength. The number, thickness, and the connectivity of plates and rods determine trabecular bone strength. Bone composition is difficult to measure non-invasively. The degree of collagen crosslinking and the density of collagen contribute to bone matrix strength. Newly formed protein matrix subsequently becomes mineralized and how the hydroxyapatite crystals are arranged within the matrix and the degree of mineralization contribute to bone hardness and strength

The determinants of bone strength are complex but can be divided into four basic components: size, shape, architecture and composition (Fig. 2). Bone has a unique ability to coordinately adjust these traits. This results in a structure that is sufficiently stiff to resist habitual loads but minimizes mass, keeping the overall energy of movement to a minimum. The overall strength of a bone depends on the proportion of cortical and trabecular tissues, their morphologies and their material properties, and the interactions among these traits. An individual’s unique genetic program also contributes to bone strength; it is estimated that up to 70% of ultimate bone strength and structure is genetically determined [11].

### Bone size

The pubertal transition is the critical period in which bone size is ultimately determined. Under the influence of androgens, the periosteum undergoes expansion resulting in a greater bone cross-sectional area [12]. Endosteal resorption occurs simultaneously but not at the rate of periosteal apposition. The end result is a larger bone and thicker cortex. Estrogens also direct an increase in periosteal expansion but not to the degree of androgens. Women generally also have less endosteal bone resorption ultimately leading to a larger bone, although smaller than in men, but the strength is maintained and compensated by a relatively thicker cortex compared to men. Overall, the relatively smaller bone size in women translates to an increased risk for fracture.

### Bone shape

Each of the 206 bones is generally well adapted to resist their habitual loads. This process, called functional adaptation, occurs primarily during growth and results in a biological system that is robust to the relatively narrow range of daily loads. Habitual loading is an important contributor to bone modeling and remodeling, and ultimately bone shape. Bone surfaces that experience the greatest compressive or tensile loads respond with increased bone mass. Conversely, skeletal unloading leads to increased bone resorption and bone loss. Daily activities result in a load (force) being applied to the bones of the skeleton, whether weight bearing or non-weight bearing. These loads cause the bone to deform with the amount of deformation being dependent on the applied load and the stiffness of the structure. A stiff structure will deform less than a compliant structure under the same load. These loads are generally small enough that the system returns to its original state when the load is removed.

Because bone is well adapted to these habitual loads, this process may leave the bone vulnerable or weak to loads applied in a different direction, such as during a fall. For example, the proximal femur is extremely strong when loaded in a direction consistent with habitual forces. A healthy femur can withstand nearly 8 kN (~ 1800 pounds) before breaking, and so theoretically two femurs should be able to support the weight of an average car. However, the strength of the proximal femur declines by more than 50% when loaded in a direction consistent with that seen during a fall to the side [13]. The mass and material properties are the same regardless of the loading direction, but the orientation of these traits relative to the two loading directions differs greatly. Under a fall-to-the-side loading direction, it is the amount of bone mass remaining within the femur that represents the resistance to fracturing.

### Bone architecture

Bone architecture, the trabecular arrangement combined with cortical bone thickness and porosity, provides a scaffold that is significantly stronger than an equal mass of solid bone. The trabecular bone scaffold within the marrow space is composed of plates and rods (Fig. 1) with a higher plate:rod ratio conferring strength. With aging, plates become more rod-like and plate connectivity with the rods declines, all of which contributes to lower bone strength and stiffness.

**Fig. 3:**
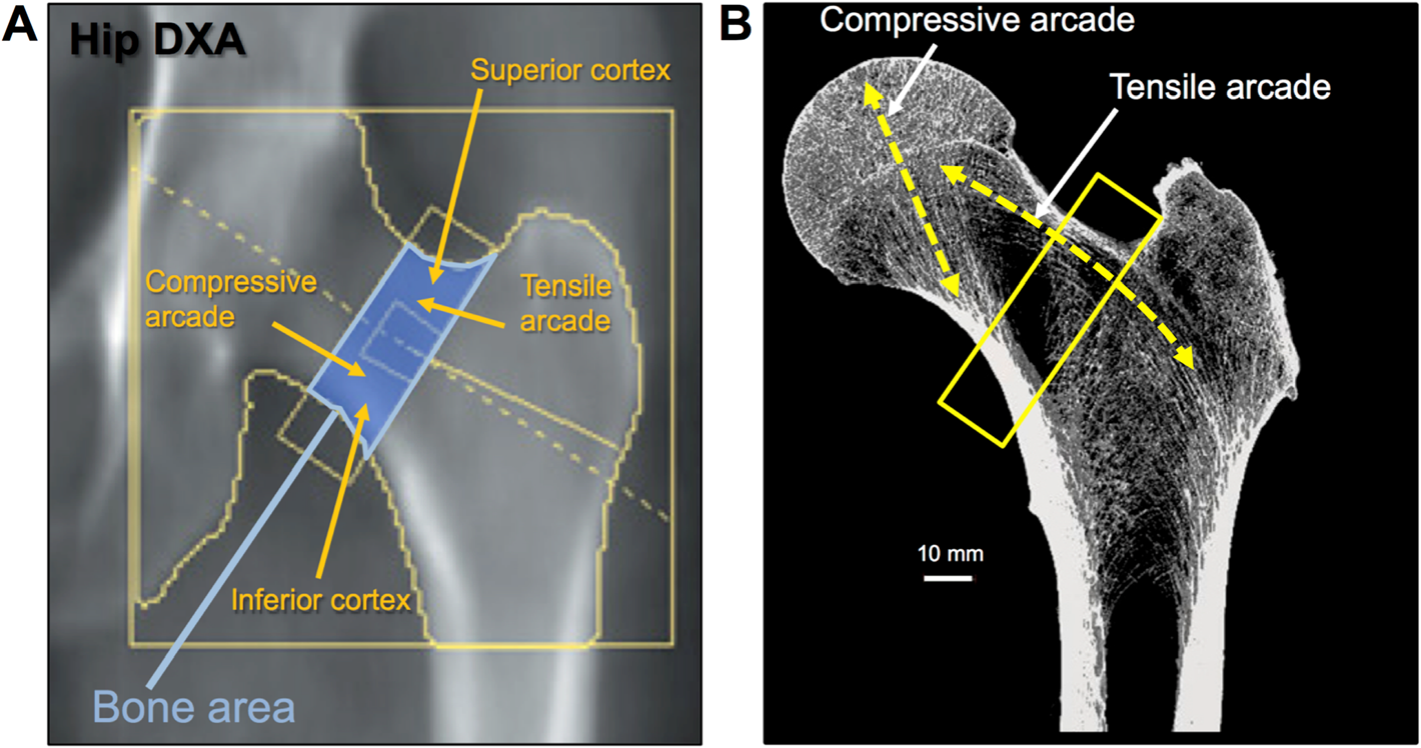
Strategic arrangement of cortical and trabecular bone. The proximal femur experiences forces in different directions. **a** The critical aspects of femoral neck strength superimposed onto a hip DXA scan image. **b** With standing, the femoral neck experiences compress forces on the inferior surface and tensile forces on the superior surface. Compressive loads are reinforced with a compressive arcade composed of a thickened inferior cortex and an additional trabecular network. The tensile arcade is reinforced with a network of trabecular bone. These reinforcements are combined with lateral and medial cortices that provide additional reinforcements against side-to-side forces. NanoCT images were taken at 27 μm resolution using a phoenix nanotom-s (GE Sensing and Inspection Technologies, GmbH, Wunstorf, Germany)

The arrangement of trabecular bone is strategic to provide maximal strength. This is especially evident in the femoral neck [14, 15]. The ability of the inferior cortex and compressive arcade to resist compressive loads, combined with the superior cortex and tensile arcade to resist tensile loads provides maximal strength and flexibility (Fig. 3). Failure of this cooperative network is the reason for femoral neck fractures. Thus, efforts to maintain strength by applying more or greater loads to stimulate bone formation may make the bone stronger for daily loads. Unfortunately, upon losing appreciable bone mass in the femur (e.g., tensile arcade), it remains unclear whether an exercise program will be able to restore lost tissue.

Cortical porosity is another layer that defines cortical strength independent of cortical size. Heightened osteoclast resorption expand existing Haversian canals, creating large macro-pores and leading to the progressive thinning of the cortical tissue that is capable of bearing load. With age, pore volume increases but pore number remains relatively constant [16]. It is mechanically fortuitous that the resorptive process begins near the endocortical surface. The proximate location of these macropores minimizes the impact on bone strength compared to pores created closer to the periosteal surface [17–19]. Despite this biomechanically favorable location of bone loss, cortical porosity is a strong predictor of fracture especially in the cortical rich area of the forearm [20]. Osteoclast resorption and resultant porosity of the trabecular bone surface also contributes to bone fragility.

### Bone composition

Bone quality was originally defined as the factors contributing to strength that are not explained by BMD. From a clinical perspective, this definition provides a name to unexplained factors. From an engineering perspective, this definition makes little sense as it does not provide a definable biomechanical pathway linking strength to physical bone traits and ultimately to the underlying biology [21]. The composition of bone that contributes to bone quality—the regular arrangement of collagen, the degree of crosslinking of adjacent collagen fibrils and mineral to protein matrix ratio—all contribute to bone quality. Diseases such as Paget’s disease, diabetes mellitus, and osteogenesis imperfecta and long-term use of glucocorticoids contribute to poor bone quality. Another example of decreased bone quality are stress fractures that occur due to repetitive damage. High bone turnover is also another component that leads to poor bone quality. Bone turnover markers have been reported to be predictive of fracture risk that is independent of BMD [22–24]. Clinical tests to assess bone quality are currently being developed but are not available for routine clinical use.

### Skeletal biomechanical changes with puberty and aging

Net bone loss or formation is dependent on the balance between bone resorption and bone formation. The net bone formation of skeletal “modeling” of childhood ensures structural support during the critical growth period. Before puberty, skeletal development is nearly identical in boys and girls. New bone formation on the periosteum exceeds endosteal bone breakdown resulting in skeletal expansion. Trabecular bone continues to develop in this period. Until the period of peak bone mass at 30-40 years of age in men and women, total skeletal bone formation is greater than resorption. With aging, and especially at menopause, this balance tips toward resorption and bone loss. After the period of peak bone mass, men and women lose similar amounts of cortical bone by endosteal resorption, but men have greater periosteal apposition than women, so that in men, net bone loss is less. Both sexes experience trabecular bone loss with aging, but this effect is more pronounced in women than in men. The decline in estrogen at menopause promotes loss of trabecular connectivity and exerts a profound impact on bone strength [25].

## Tools currently available to assess fracture risk

### DXA

Despite its underappreciated limitations, DXA is often considered to be the gold standard imaging test for diagnosing osteoporosis. Unlike older DXA machines that employed higher radiation, today’s scanners emit significantly less radiation per scan—as little as 1-10 microsieverts (μSv) with about 7 μSv being the average ionizing radiation dose received from natural background radiation [26]. DXA is widely available in hospital and outpatient practices. By utilizing two different energies this technology is able to differentiate the mineralized bone, composed of hydroxyapatite, from soft tissues such as skin, fat and muscle. The two X-ray energies differ in their attenuation profiles after passing through bone and soft tissues. Older DXA units employed a pencil beam that had a limited scan area resulting in longer scan times. Modern DXA units use a fan-shaped beam that translates to scan times of 10-30 s. Standard DXA scans report bone density in grams/cm^2^ and is derived from dividing the bone mineral content (BMC) in grams by the region of interest (ROI) scanned in cm^2^.

The standard locations for DXA measurement are the L1-L4 lumbar spine, hip, and forearm. These reference locations were originally selected because morbidity from fractures at these locations is high, especially at the spine and hip. DXA results are reported as the standard deviation (SD) from a population mean, comparing the subject to a population at peak bone mass (T-score) and to an age-matched population (Z-score). One and two SDs from the mean encompass 68 and 95% of a population, respectively. Since peak bone mass occurs at between 30 and 40 years of age, it is appropriate to use Z-scores in children and young adults who have yet to achieve peak bone mass.

The distribution of bone density across a population is dependent on race, age and gender. For example, African-Americans have lower rates of fracture compared to US Caucasians and Asians and this parallels the population distribution differences among races [27]. In one study, the age-adjusted mean for femoral neck BMD was 0.686 g/cm2 in US Caucasians and 0.841 g/cm2 in African Americans [28]. Because of such racial and ethnic differences, the significance of T-scores must be considered based on the fracture risk of ethnic and racially matched persons. A similar rationale can be applied to men who have larger skeletal structures compared to women. To control for racial differences, DXA calculates T-scores using normative databases based on NHANES III data that include non-Hispanic White, Black, Hispanic and Asian individuals [29]. A pediatric normative base is also available.

As stated before, bone size is directly related to strength. DXA does not account for bone size in assessing fracture risk. Attempts to correct bone size for height and weight have been reported [30]. Some DXA manufacturers allow for weight correction in the calculation of Z-scores to adjust for an expected decrease in fracture risk as weight increases. Height correction is especially important in assessing fracture risk in children affected by short stature or growth delay [31].

DXA images are a 2-dimensional (vertical and horizontal) condensation of a 3-dimensional structure. As such, bone thickness is not measured in this scan. The BMC measured reflects the amount of cortical and trabecular tissue present within a structure that acts to attenuate the X-ray signal; bones with more tissue attenuate the signal to a greater degree resulting in a higher gray value and BMC measure. Bone area is a measure of the size of the ROI. For the hip, the ROI width is fixed and thus variation in bone area reflects differences in external bone size. The ratio of these two variables provides a measure of the mass density but not a measure of morphology or material properties. Further, BMD does not differentiate whether the variation in BMD arises from differences in cortical mass, trabecular mass, or external bone size.

**Fig. 4:**
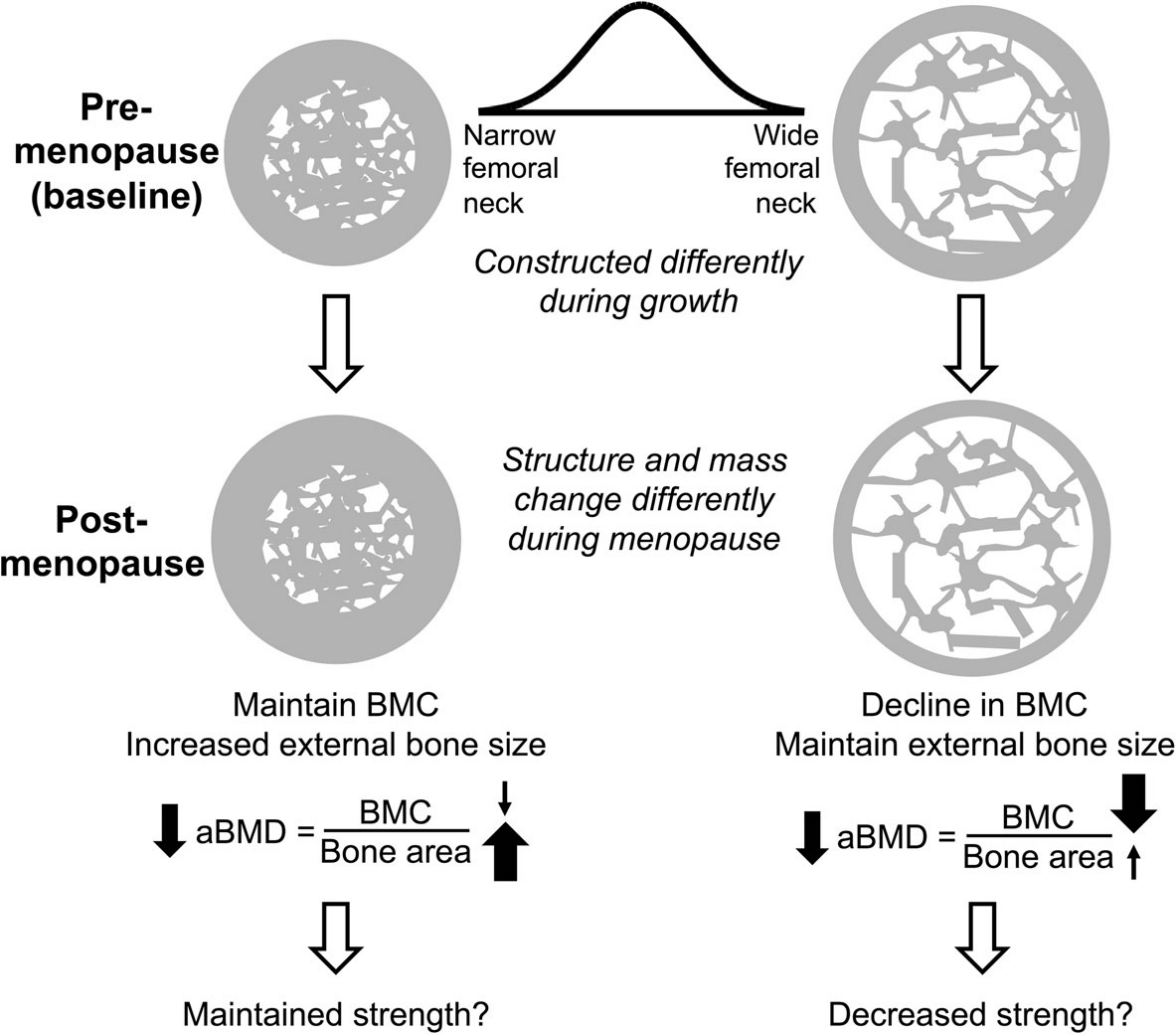
Areal BMD as determined by DXA declines with aging for different reasons. With aging, women with smaller femoral necks tend to increase bone area through an increase in cortical thickness by an increase in periosteal and endosteal bone formation. Since BMD may only decrease slightly but bone area increases more, the result is lower areal BMD as measured by DXA despite likely having little change in bone strength. In the case of women with larger femoral necks, the endosteal cortex undergoes excessive resorption without periosteal expansion resulting in a thinner cortex. The result is a lower BMC without significant change in bone area. The DXA areal BMD decreases and may result in a bone with less strength. Adapted from Jepsen, et al. JBMR 2017 [32]

Conventional wisdom is that women uniformly lose endosteal and trabecular bone in a similar pattern. Recent data however suggest that the pattern of bone loss with aging in women is not uniform [32]. Bone shape and size at the menopause transition may in fact have a critical role in determining long-term bone loss with aging. Women with narrower femoral necks experienced modest decreases in BMC compared to those with wider femoral necks (Fig. 4). But, women with narrow femoral necks also had larger increases in femoral neck area compared to women with wider femoral necks. BMD is the quotient of the BMC divided by the area. Because the larger increase in the denominator (area) in women with narrow femoral necks is similarly matched by the larger decrease in the numerator (BMC) in women with wide femoral necks, the result is that both groups have similar losses in BMD over time but for very different reasons. The impact of these structural and mass changes on strength is currently under investigation. In addition to the previous discussion regarding how most fragility fractures occur in persons with T-scores > − 2.5, this example illustrates another limitation of DXA scanning to accurately predict bone strength and fracture risk.

### Trabecular bone score (TBS)

Other than BMD, fracture risk is dependent on bone geometry, microarchitecture, microdamage, rate of bone turnover, and mineralization—all of which contribute to bone strength. TBS indirectly assesses skeletal texture using DXA images and can be used to predict the risk of spine and hip fractures in women and men above the age of 40. It has been validated in multiple cohorts with large numbers of subjects and shown to improve fracture risk prediction beyond that obtained by DXA. TBS is available for clinical use in the United States [33].

The TBS is a textural index based on evaluating the pixel gray-level variations in the lumbar DXA image [34]. Well-structured bone produces 2-D DXA images that are homogenous with small gray-value amplitude variations. On the other hand, bone that is of poor quality produces higher gray-value amplitudes. TBS is a unitless calculation of the sum of the squared gray-level differences between pixels at a specific distance. The steeper slope represents well-structured bone while the lower slope is suggestive of poorer architecture. Based on values provided by the manufacturer, TBS > 1.350 is normal, 1.200-1.350 is consistent with partially degraded bone and < 1.200 indicates degraded bone.

TBS is typically measured at the L1-L4 lumbar spine (LS), the same sites used for DXA. The results are provided for each vertebral body as well as the composite for L1-L4. Unlike DXA, osteoarthritic changes have little impact on data generated by TBS.

Several studies have shown that TBS predicts clinical, hip and vertebral fractures in postmenopausal women [35, 36]. Some longitudinal studies have reported that TBS predicts fracture risk in men over the age of 40 but data on premenopausal women are lacking [37, 38]. In addition, a meta-analysis of 14 prospective population-based cohorts reported that TBS provided additional information on the 10-year fracture probability as estimated by the standard FRAX tool [39].

A new feature is available on the online FRAX risk assessment tool with an option to “adjust with TBS”. A low TBS would increase the FRAX risk of major osteoporotic fracture by 1.5-1.6 fold [40]. Changes in TBS are much smaller than LS BMD with osteoporosis treatment and therefore the role of using TBS to monitor patients on therapy is uncertain. There is no data on the impact of a change in TBS on fracture risk.

### Quantitative US (QUS)

QUS can provide information on bone structure and fragility. Due to its use of low-frequency ultrasound it is safe and a relatively inexpensive method to assess for osteoporosis. The two main parameters measured are the velocity of sound (VOS) and broadband US attenuation (BUA). Data provided by QUS of the heel have been shown to correlate with the risk of fracture [41] but it is not used routinely for diagnosis of osteoporosis.

### Quantitative CT (QCT)

QCT provides volumetric 3D measurements by utilizing a low dose scan protocol and offers adequate details of the cortical and trabecular bone to generate reasonable estimates of strength through engineering-based analyses such as finite element analysis (FEA) and probabilistic modeling. QCT is most commonly studied at the lumbar spine and hip. A variation of QCT, high-resolution peripheral quantitative computed tomography (HR-pQCT), is mostly used to assess tibia and radius bone architecture and density. The associations between HR-pQCT-based vertebral bone measurements and prevalent vertebral fractures depend on the spinal locations of both bone measurement and fracture [42, 43]. An unclear correlation between QCT and other non-vertebral osteoporotic fractures along with higher exposure to ionizing radiation and cost have resulted in an infrequent use of these scans. In addition, large precision errors with repeat measurements and unclear methods to adjust for variation in marrow fat and soft tissue density remain challenges for wider clinical use of QCT.

### Fracture risk assessment calculators

Until recently, treatment decisions were made primarily using T-scores but the over-reliance on this score has resulted in over-treatment, especially in younger patients who may in fact have a lower fracture risk. Clinical risk factors, such as age, previous fragility fracture, parental history of hip fracture at age < 80, smoking, excessive alcohol intake, and prolonged glucocorticoid use, all have been shown to confer risk independent of BMD measurement. Using these risk factors and BMD data, fracture prediction algorithms have been developed.

The FRAX scoring system (https://www.sheffield.ac.uk/FRAX/) is one such fracture prediction algorithm [44]. It is the most widely used fracture prediction algorithm. The score provides a 10-year probability of having a hip or major osteoporotic fracture with or without data on femoral neck BMD. The algorithm has been well validated in independent cohorts [45]. The Garvan calculator is another tool used to predict fracture risk (https://www.garvan.org.au/promotions/bone-fracture-risk/calculator/). The calculator was developed using data obtained from the Dubbo Osteoporosis Epidemiology Study at Sydney’s Garvan Institute. In addition to demographic variables and BMD or T-scores, the Garvan calculator takes into account the number of falls. The tool has been validated and is found to be clinically useful in predicting fractures in those at high risk [46]. Other calculators such as Osteoporosis Canada and FORE FRC v 2.0 predict the 10-year fracture risk but are not commonly used. The Male Osteoporosis Risk Estimation Score (MORES) was reported to be a better tool to predict hip osteoporosis in men compared to FRAX [47]. While acknowledging that these calculators do not include all risk factors and can underestimate the fracture risk, they serve as a valuable tool to assist physicians in assessing risk with one long-term goal of avoidance of treatment in patients at low fracture risk [48].

### Bone turnover markers (BTMs)

BTMs are released during bone remodeling and can be measured in blood or urine. BTMs provide an assessment of bone remodeling rate and are surrogate end-points for fracture, bone quality and effectiveness of the therapy. They are grouped into two broad categories: bone resorption and bone formation markers. Collagen degradation products, namely C-terminal cross-linked telopeptide of type 1 collagen (βCTX), are released during bone resorption and reflect osteoclast activity. Bone formation markers such as procollagen type I N-terminal propeptide (PINP) and procollagen type I C-terminal propeptide (PICP) are peptides derived from posttranslational cleavage of type I procollagen molecules by proteases at the N- and C-terminus, respectively. These markers reflect osteoblast function and activity.

Commercially available βCTX assays have been developed with low method-specific difference and inter-assay variability. βCTX itself demonstrates significant variation due to circadian rhythm and food intake. It is best measured in the fasted state and in the morning. The International Osteoporosis Foundation recommends using PINP and βCTX to assess bone formation and bone resorption, respectively [24].

The utility of bone turnover markers in assessing the risk of fracture has been studied in postmenopausal women. In the OFLEY cohort, healthy postmenopausal women who had BTMs in the highest quartile were noted to have a two-fold increase in the risk of fractures with a RR of 1.8% [49]. In another cohort of older postmenopausal women, high levels of osteocalcin (bone formation marker) were associated with a higher risk of fractures [50]. BTMs can also be used for monitoring osteoporosis treatment. In the IMPACT study, greater than 30% decline in the level of urine NTX was associated with a 50% reduction in non-vertebral fractures [51]. In postmenopausal women treated with teriparatide, an increase in P1NP at three months correlated with an increase in LS BMD at 18 months [52].

While there has been widespread use of these markers for monitoring therapy in osteoporosis, treatment goals based on fracture reduction have not been defined. In addition, there is insufficient data on the use of bone turnover markers for diagnosis of osteoporosis, identifying candidates for treatment, and determination of the length of bisphosphonate “drug holidays”.

## Treatment-related changes in bone density and architecture

Antiresorptive and anabolic therapies increase spine and hip BMD, with the highest increases in the spine (Table 1). As newer agents are studied, a trend in more efficacious BMD improvement with each new agent is apparent. Although many osteoporosis treatments have not been directly compared in head-to-head trials, the mechanisms of actions of these newer treatments often predict a superior efficacy in increasing BMD.

**Table 1:** Summary of treatment-related changes in human skeletal architecture. Only published studies that reported defined skeletal architectural indices were included in the Table

	Areal BMD		HR-pQCT, QCT				QCT			Bone biopsy/QCT		
Location	Spine	Hip	Radius/Tibia				Spine			Hip		
Measure	BMD (Approx. % increase)		Per.Diam	CoPo	CtTh	Tb	CoPo	CtTh	BV/TV	CoPo	CtTh	BV/TV
Bisphosphonates	4^(a)^	2-2.5^(a)^		**↓**,NS^(g)^	**↑** ^(g)^	**↑**,NS^(g)^				**↓** ^(m)^		NS^(m)^
Denosumab	5.5^(b)^	3^(b)^		**↓**,NS^(h)^	**↑** ^(h)^	**↑** ^(h)^				**↓** ^(n)^		
Teriparatide	9^(c)^	3^(c)^	**↑** ^(f)^	**↑**,NS^(i)^	**↑**,NS ^(i)^	**↑**,**↓**^(i)^		NS^(j)^	**↑** ^(k)^	**↑** ^(0)^	**↑** ^(p)^	**↑** ^(p)^
Abaloparatide	11^(d)^	4^(d)^										
Romosozumab	13.5^(e)^	6.5^(e)^						**↑** ^(l)^	**↑** ^(l)^			**↑** ^(l)^

*BMD* bone mineral density, *Per.Diam* periosteal diameter, *CoPo* cortical porosity, *CtTh* cortical thickness; *Tb* trabecular indices; *BV/TV* bone volume/tissue volume, *NS* not significant, *HR-pQCT* high-resolution peripheral quantitative computed tomography, *QCT* quantitative computed tomography

Notes:

a. 12 months of treatment [53, 54, 56, 81, 82]

b. 12 months of treatment [65]

c. 18 months of treatment [66]

d. 18 months of treatment [67]

e. 12 months of treatment [68, 69]

f. [71]

g. Cortical volumetric BMD (Ct vBMD) as a surrogate for CtPo, Tb = Tb vBMD [83]; CtTh significant only for tibia, Tb vBMD increased at tibia [84]; Ct vBMD as a surrogate for CtPo with difference only in tibia [85, 86]

h. CoPo as a surrogate for Ct vBMD, Tb as a marker of trabecular volumetric BMD (Tb vBMD) [83]; [70, 87]

i. 24 months of treatment [70]; 18 months of treatment, increase in plate Tb number and thickness [88]; 18 months of treatment, increase in trabecular number [89]; 18 months of treatment, increase in CtTh in tibia only, reduction in trabecular thickness [90]

j. [91]

k. [92]

l. [91, 93]

m. [94]

n. [95]

o. [96]

p. [97]

All approved osteoporosis medications produce significant increases in spine and hip BMD as measured by DXA. The degree of BMD increase in the spine is likely a consequence of the greater surface area of trabecular-rich vertebral bodies on which the agents act. Twelve months of treatment with bisphosphonates increased BMD by approximately 4% in the spine and 2% in the hip as reported in the landmark FIT, VERT, BONE, and Horizon trials [53–56]. The efficacy of daily, weekly and monthly oral and yearly IV bisphosphonate medications are similar [57–61]. Compliance with oral bisphosphonates is a common factor in those patients who fail to respond to treatment [62–64]. Denosumab has even greater effects likely owing to its enhanced ability to suppress bone resorption [65]. Teriparatide, an anabolic agent, increases spine and hip BMD [66]. Abaloparatide, another recently available anabolic agent, also markedly increases spine and hip BMD [67].

Romosozumab, not yet approved for treatment, is a humanized monoclonal antibody that targets sclerostin, and has been reported to increase spine BMD approximately 13.5% and hip BMD approximately 6.5% after 12 months of treatment [68, 69].

**Fig. 5:**
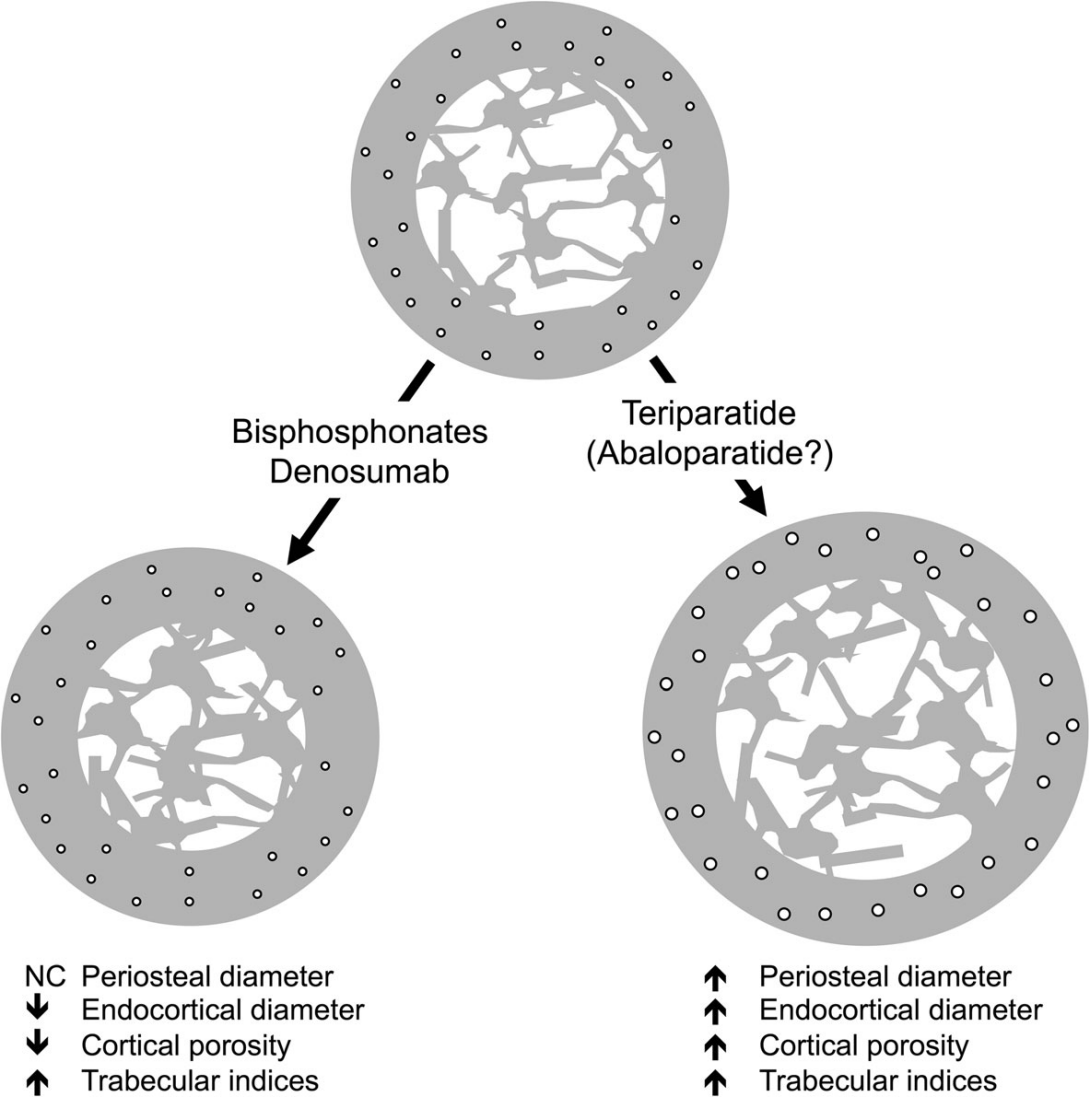
Structural changes in bone with osteoporosis medications. The anti-resorptive medications (bisphosphonates and denosumab) and anabolic medications (teriparatide and likely abaloparatide) produce very different structural changes in bone. Although both classes increase trabecular bone, their effects on cortical bone are different. Bisphosphonates and denosumab do not expand periosteal bone but do decrease the endosteal diameter by an increase in endosteal bone volume. Anti-resorptives also reduce cortical porosity. Anabolic agents lead to an increase in periosteal bone with a simultaneous increase in endosteal bone resorption resulting in a bone without a large change in cortical thickness. At the same time, anabolic agents increase cortical porosity. Despite the increase in cortical porosity, the larger bone has increased strength. NC = no change

Numerous published studies have reported the architectural changes in the skeleton with such agents using a variety of techniques that include HR-pQCT and QCT of in situ hip and spine as well as similar techniques of iliac crest bone biopsy samples. What has become clear is that they do not uniformly produce similar results (Table 1, Fig. 5). Bisphosphonates increase cortical thickness primarily by decreasing the endosteal perimeter, partially through the filling in of previously excavated resorption pits at the endosteal surfaces. In addition, bisphosphonates also reduce cortical porosity and increase the amount of trabecular bone. Denosumab has similar effects and presumably to a higher degree owing to its improved fracture reduction compared to bisphosphonates.

Teriparatide has unusual effects on cortical bone. While spine and hip BMD increased with this agent, forearm BMD declined prompting a closer inspection of architectural changes in cortical-rich areas [66]. Teriparatide increases cortical porosity through two not mutually exclusive mechanisms: 1) increased osteocyte-directed bone resorption and 2) enhanced cortical periosteal bone expansion that leaves a larger proportion of under-mineralized bone [70–72]. Furthermore, since the denominator in the BMD calculation is bone area and teriparatide certainly causes an increase in periosteal diameter compared to BMC, BMD expectedly decreases. Presumably abaloparatide has similar effects but such detailed human architectural analyses have not been published. Romosozumab is reported to increase cortical thickness and trabecular bone volume, but how this agent affects cortical porosity and bone size has not been published.

## The future of goal-directed therapy

Goal-directed treatment for osteoporosis has been advocated as a superior strategy rather than treatment decisions made solely on DXA T-scores [73]. Rather than arbitrary recommendations to treat osteoporosis for 5 or 10 years with oral bisphosphonates or 3 to 6 years with IV bisphosphonates, depending on T-scores or whether a patient is deemed either low or high risk for fracture, treatment length should ideally be based on achieving a particular fracture risk threshold [74]. The FRAX risk stratification system has raised awareness among clinicians that other strong risk factors for fracture exist other than DXA T-scores—age, previous fragility fracture, high fall risk, long-term glucocorticoid use and other diseases associated with high fracture risk that include diabetes mellitus. However, neither bone size nor architectural makeup is routinely measured but clearly have large impacts on bone strength. For example, the femoral neck of two individuals could have the same BMD but the structure of these could be vastly different owing to the differences in size with a smaller femoral neck possessing lower strength. The bone area is already routinely reported in DXA scans but is not routinely utilized to assess risk. However, recent data support that bone size is dynamic and that postmenopausal women with smaller femoral neck size may in fact be at lower risk for fracture as they age compared to women with larger femoral neck size due to adapted changes with aging [32]. How these inter-individual differences in the age-changes of structure and mass affect bone strength and fracture has yet to be fully determined.

New imaging techniques that not only measure BMD but also measure critical indices directly related to fracture risk such as bone size, porosity, cortical thickness, trabecular volume and the mineral to matrix ratio are needed. Even better, having such a device that is affordable and appropriately sized allowing clinicians to assess fracture risk in the clinic is the future of osteoporosis care. Until the radiation dose of QCT is lower, such imaging modalities are not practical for routine screening and treatment monitoring. Methods to directly measure bone quality such as reference point indentation are investigative. This method is limited by pain, differing outcome measures amongst cohorts [75–77] and are inconsistently related to traditional tissue-level mechanical properties [78, 79]. Compact ultrasound imaging devices that measure forearm cortical bone size and trabecular bone density is an exciting new area [80].

With advancing imaging methods, we can envision a treatment strategy whereby osteoporosis medications are selected based on individual skeletal characteristics. For example, patients with larger bones, and thinner and porous cortices may benefit from bisphosphonates and denosumab, to reduce endocortical resorption that would ultimately increase cortical thickness. Conversely in patients with smaller bones whose cortex is not especially porous, teriparatide or abaloparatide may provide maximal bone strength. Clearly, this is an area of further research.

## Conclusions

The challenges to wider clinical utilization of biomechanical traits in treatment decisions involve 1) better understanding of biomechanical principles, 2) developing an appreciation of how bone strength depends on multiple traits, 3) incorporating the concept that people fracture for different biomechanical reasons, and 4) coalescing this information into a digestible outcome parameter that can be used clinically are areas where more work is needed. Using these sophisticated technologies, clinicians will be able to select therapy that targets skeletal characteristics. While much work remains in redefining and identifying individuals at risk of fractures, updating the current system of diagnosis and generating new technologies, we inch closer to the future of osteoporosis care and personalized medicine.

## Abbreviations

BMC: Bone mineral content
BMD: Bone mineral density
BTM: Bone turnover marker
DXA: Dual-energy X-ray absorptiometry
HR-pQCT: High-resolution peripheral quantitative computed tomography
LS: Lumbar spine
P1CP: Procollagen type I C-terminal propeptide
P1NP: Procollagen type I N-terminal propeptide
QCT: Quantitative computed tomography
QUS: Quantitative ultrasound
ROI: Region of interest
SD: Standard deviation
TBS: Trabecular bone score
WHO: World Health Organization
βCTX: C-terminal cross-linked telopeptide of type 1 collagen
μSv: Microsieverts
